# Abstracts

**DOI:** 10.1155/S1463924682000030

**Published:** 1982


Journal of Automatic Chemistry, Volume 4, Number 1 (January–March 1982), pages 3–5

### Abstracts

### Page 7

### An evaluation of the IL 508 eight-channel blood-chemistry analyser

R. W. Logan *et al.*

An evaluation of the new IL 508 is described. The instrument is a microprocessor-controlled eight-channel analyser requiring a plasma volume of 230 $\mu$l. The instrument has a visual display unit and a thermal printer for the recording of results. There is also an optional output for interfacing to either a computer or a line printer. The instrument operates at a speed of 100 samples/h and has a stat capability for introducing an emergency sample.

Results are presented for carry-over, linearity, within-batch and between-batch precision and accuracy. From these results the IL 508 would appear to serve a useful function in the clinical chemistry laboratory.

### Une évaluation de l'analyseur de sang à huit canaux IL 508

R. W. Logan *et al.*

Une évaluation du nouveau IL 508 est décrite. Cet instrument est un analyseur à huit canaux piloté par un microprocesseur et demandant un volume de plasma de 230 $\mu$l. L'instrument se compose d'une unité d'affichage visuel et d'une imprimante thermique pour enregistrer les résultats. Comme option, une sortie de données est disponible pour le raccordement ou d'un ordinateur ou d'une imprimante à lignes. La capacité de l'instrument est d'une fréquence de 100 échantillons par heure et grace à la fonction 'stat' des échantillons d'urgence peuvent être inserés.

Des informations sont données pour la contamination, la linéarité, la précision et la réproductibilité dans une série et entre deux séries. A base de ces résultats, le IL 508 semble être capable de remplir une fonction utile dans le laboratoire chimique d'une clinique.

### Eine Evaluation des IL 508 Acht-Kanal Blutanalysators

R. W. Logan *et al.*

Es wird eine Evaluation des neuen IL 508 beschrieben. Das Instrument ist ein mikroprozessor-gesteuerter Acht-Kanal Analysator, der ein Plasmavolumen von 230 $\mu$l erfordert. Das Instrument verfügt über eine Bildschirmeinheit und einen thermischen Drucker für die Aufzeichnung der Resultate. Als Option steht ein Datenausgang zur Verfügung zur Verbindung mit einem Computer oder einem Zeilendrucker. Das Instrument arbeitet mit einem Durchsatz von 100 Proben pro Stunde und verfügt über die Möglichkeit Eilproben einzuführen.

Es werden Daten angeführt für die Verschleppung, Linearität sowie Genauigkeit und Wiederholbarkeit innerhalb einer Serie und von Serie zu Serie. Auf Grund dieser Resultate scheint der IL 508 eine nützliche Funktion im klinisch-chemischen Laboratorium übernehmen zu können.

---

### Page 11

### Immobilized uricase for automated assay of uric acid in serum

L. P. León *et al.*

Uricase (urate oxidase, EC 1.7.3.3.) immobilized at the inner wall of polyamide tubing was used in continuous flow analysers to quantitate uric acid in serum. Concentration change of 1 mg/dl for the analyte in the sample yields a change of $3 \times 10^{-2}$ absorbance units in the outport and carry-over does not exceed 5%. The linearity of the method is good for uric acid concentration up to 15 mg/dl and the results correlate well with those obtained by the ultra-violet spectrophotometric reference method. The operational stability of the enzyme coil is sufficient to allow continuous flow analysis of at least 10 000 serum samples equally, or better than current uric acid methods using free uricase in solution.

### Uricases fixées pour l'analyse automatisée d'acide urique dans un sérum

L. P. Leon *et al.*

Uricases (urate oxidase, EC 1.7.3.3.) fixées à la face interne des tuyaux polyamides ont été utilisées pour la détermination de l'acide urique d'un sérum dans des analyseurs à flux continu. Un changement de concentration de 1 mg/dl de la substance à déterminer dans l'échantillon cause un changement de $3 \times 10^{-2}$ unité d'absorption à la sortie. La contamination ne dépasse pas 5%. La linéarité de la méthode est bonne pour les concentrations d'acide urique jusqu'à 15 mg/dl. Les résultats sont en bonne corrélation avec ceux obtenus par la méthode de référence spectrophotométrique ultraviolet. La stabilité de fonctionnement du tube à enzymes suffit à analyser au moins 10 000 échantillons de sérum dans le flux continu d'une manière égale ou même meilleure que les méthodes d'acide uriques existantes, utilisant de l'uricase libre en solution.

### Inmobilisierte Urikase für die automatisierte Analyse von Harnsäure in Serum

L. P. León *et al.*

Urikase (urate oxidase, EC 1.7.3.3.) die auf der Innenwandung von Polyamid Schläuchen inmobilisiert war, wurde für die quantitative Bestimmung von Harnsäure in Serum in Analysatoren mit kontinuierlichem Fluss benützt. Eine Konzentrationsänderung von 1 mg/dl der zu bestimmenden Substanz in der Probe bewirkt eine Aenderung von $3 \times 10^{-2}$ Absorptionseinheiten am Ausgang. Die Verschleppung bleibt unterhalb von 5%. Die Linearität der Methode ist für Harnsäurekonzentrationen bis zu 15 mg/dl gut, und die Resultate korrelieren gut mit denen, die mit einer ultraviolett spektrophotometrischen Referenzmethode erhalten wurden. Die Betriebsstabilität der Enzymschlange ist genügend um mindestens 10 000 Serumproben im kontinuierlichem Fluss gleichwertig oder besser als mit bestehenden Harnsäuremethoden, die freie Urikase in Lösung benützen, zu analysieren.

---

### Page 17

### Direct flow automated serum-ion determination

F. Ceriotti and P. Bonvicini

A direct, specific and sensitive automated-flow method for serum-iron determination using ferrozine as the chromogen is described. Usually in this approach, dialysis is necessary and presents difficulties; it has been avoided here by exploiting the possibility of eliminating turbidity, without addition of surfactants, by working in dilute hydrochloric acid and of preventing the interference of copper by adding thiosemicarbazide. Some problems related to adsorption of iron from aqueous solutions on tygon tubing have been investigated and solved.

### Détermination direct du fer d'un sérum flux en continu

F. Ceriotti et P. Bonvicini

Une méthode directe, automatisée, spécifique, sensitive et dans le flux est décrite avec laquelle le fer d'un sérum se détermine en utilisant de la ferrozine comme agent de coloration. Normalement, cette méthode exige la dialyse, rendant difficile la détermination de fer. Ici, les difficultées sont évitées en utilisant le fait que, en travaillant avec acide chlorhydrique dilué, le trouble peut être éliminé sans additionner des substances tensio-actifs. En outre, l'interférence par le cuivre s'évite en additionnant du thiosémicarbacide. Quelques problèmes relatifs à l'absorption de fer des solutions aqueuses aux tuyaux tygone ont été examinés et résolus.

**Direkte Bestimmung von Eisen in Serum im Probenstrom**

F. Ceriotti und P. Bonvicini

Die Arbeit beschreibt eine direkte, spezifische und empfindliche, automatisierte Methode für die Serum-Eisenbestimmung im Probenstrom unter Benützung von Ferrozin als Farbträger. Normalerweise wird bei dieser Methode Dialyse notwendig, die für die Eisenbestimmung Schwierigkeiten verursacht. Dies wird hier vermieden durch die Ausnützung der Möglichkeit, das bei Arbeit in verdünnter Salzsäure die Trübung ohne die Beimengung von oberflächenaktiven Substanzen eliminiert werden kann und das durch die Beimischung von Thiosemikarbazide die Störung durch Kupfer vermieden wird. Einige Probleme die im Zusammenhang mit der Absorption von Eisen aus wässerigen Lösungen an Tygon Schläuchen stehen, wurden untersucht und gelöst.

---

### Page 21

**Assessment of the Cobas Bio centrifugal analyser**
R. Bais *et al.*
The Cobas Bio centrifugal analyser incorporates dispensing and analysing systems in the unit. An Intel 8080A microprocessor controls the total system and there is space for 30 programs. The direction of the light-path is parallel to the centrifugal force so that the calculation factor is dependent only on the sample volume. The precision and accuracy of the instrument proved to be acceptable, although a number of minor modifcations to the system were suggested which, if carried out, would improve the flexibility of the Cobas Bio and increase its use in both routine and research investigations.

**Appréciation de l'analyseur à centrifuge**

R. Bais *et al.*

L'analyseur centrifuge Cobas Bio comprend des systèmes de dosage et d'analyse. Le microprocesseur Intel 8080A commande l'ensemble du système et, il y a place existante pour 30 programmes. La direction du rayon de lumière est parallèle à la force centrifuge, ainsi le facteur à calcul ne dépend que du volume de l'échantillon. L'instrument offre une réproductibilité et une précision acceptable. Cependant, un petit nombre de mofidications a été proposé qui, une fois réalisés, amélioreront la flexibilité du Cobas Bio et augmenteront son utilité dans les deux domaines: la routine et les recherches scientifiques.

**Beurteilung des Cobas Bio Zentrifugal Analysators**
R. Bais *et al.*
Der Cobas Bio Zentrifugal Analysator umfasst Dosier- und Analysen systeme. Ein Intel 8080A Mikroprozessor steuert das gesamte System, und es ist Platz vorhanden für 30 Programme. Die Richtung des Lichtstrahls ist parallel zur Zentrifugalkraft so das der Berechnungsfaktor nur vom Probenvolumen abhängt. Die Wiederholbarkeit und Genauigkeit des Instruments stellte sich als genügend heraus, obwohl eine Anzahl kleinerer Modifikationen am System vorgeschlagen wurden welche, falls sie implementiert würden, die Flexibilität des Cobas Bio verbessern und seine Nützlichkeit für Routine- und Forschungsuntersuchungen erhöhen würde.

---

### Page 25

**Program in BASIC to combine the data from two channels of an integrator, and its use in the calculation of residues per 1000 residues for amino-acid analyses**
K. J. Cronin *et al.*
A program in BASIC is reported which serves to bring together data from two channels of an integrator (Trilab 3) prior to arithmetical manipulation. The application of the method to amino-acid analysis is described; results are expressed as residues/1000 residues, but with minor modification the program could be used to combine data from two gas-liquid chromatography runs. The program includes a routine to ignore blocks of data, and another routine which allows peaks to be reassigned under operator control.

**Programme BASIC pour la combinaison des données de deux canaux d'un intégrateur, et son emploi pour le calcul des résidus par 1000 résidus dans l'analyse d'amino-acides**

K. J. Cronin *et al.*

Un programme en BASIC est présenté, destiné à la prise simultanée des données de deux canaux d'un intégrateur (Trilab 3) avant le calcul arithmétique. L'application de cette méthode pour l'analyse d'amino-acides est décrite. Les résultats sont exprimés en résidus par 1000 résidus. Par une simple modification, le programme pourrait également être utilisé pour combiner des données de deux analyses de chromatographie en phase gazeuse. Le programme comprend aussi un chapitre qui permet d'ignorer des blocs de données et un autre chapitre qui permet de réorganiser l'ordre des peaks sous le contrôle de l'opérateur.

**BASIC-Programm für die Kombination von Daten zweier Kanäle eines Integrators, und seine Benützung für die Berechnung von Residuen pro 1000 Residuen in der Analyse von Aminosäuren**
K. J. Cronin *et al.*
Es wird ein BASIC-Programm vorgestellt das dem Zusammenbringen der Daten zweier Kanäle eines Integrators (Trilab 3) vor der arithmetischen Verarbeitung dient. Die Applikation dieser Methode auf die Analyse von Aminosäuren wird beschrieben; die Resultate werden als Residuen pro 1000 Residuen ausgedrückt. Mit kleineren Modifikationen könnte das Programm auch für das Zusammenführen von Daten zweier gasflüssigchromatographie Analysen verwendet werden. Das Programm umfasst auch einen Abschnitt um Datenblöcke zu überspringen und einen andern Abschnitt, welche unter Benützerkontrolle die Neuzuordnung von Peaks erlaubt.

---

### Page 32

**Data handling on a sequential multichannel analyser computerized (SMAC): using a low-cost minicomputer**
R. Dugdale *et al.*
A low cost minicomputer system is described for use with an 'SMAC' (sequential multichannel analyser computerized), which enables real-time monitoring of the quality assurance data, storage of all patient information from the L.I.S. output, and yet allows continuous reporting of patient results as they are produced. The system can be used 'off-line' to generate a numerical (I.D.) listing of all patients results, daily quality-assurance summaries, and selected patient populations if required.

### Traitement des données pour un analyseur automatisé à canaux multiples à séquences (SMAC): Utilisation d'un ordinateur bon marché

R. Dugdale *et al.*

Un système d'un micro-ordinateur de coût avantageux est décrit. Le système est utilisé avec un analyseur automatique à multiples canaux à séquences (SMAC) et permet la surveillance en temps réel des données et contrôle de la qualité, et le stockage de toutes les informations relatives aux patients dès la sortie des données L.I.S. Cependant, toutes les informations sur les patients sont enrégistrées dès qu'elles sont produites. Le système peut être utilisé 'off-line' pour établir une liste numérique (I.D.) de tous les résultats des données relatives aux patients, un résumé journalier de contrôle de la qualité et pour sélectionner certains groupes de patients selon la nécessité.

### Datenverarbeitung für einen automatisierten sequentiellen Vielkanal Analysator (SMAC): Verwendung eines kostengünstigen Minirechners

R. Dugdale *et al.*

Die Arbeit beschreibt ein kostengünstiges Minirechner-System, das mit einem SMAC (sequential multichannel analyser computerized) benützt werden kann und welches die Echtzeitüberwachung von Qualitätsdaten und die Abspeicherung aller Patienteninformationen vom L.I.S. Ausgang erlaubt. Und trotzdem werden kontinuierlich die Patientenresultate aufgezeichnet sobald sie vorliegen. Offline kann das System benützt werden um eine numerische (I.D.) Liste mit allen Patientenresultaten auszudrücken, eine Zusammenfassung von täglichen Qualitätsdaten zu erstellen sowie ausgewählte Patientengruppen zusammenzustellen.

### Page 35

### Two-point kinetic determination of uric acid in serum with uricase, catalase and aldehyde dehydrogenase on the Kone CD analyser

G. A. Harff and F. Lamie

Two common methods for the enzymatic determination of uric acid by Haeckel's new ALDH method are investigated for their applicability to routine use on the Kone CD analyser. Only one reagent (the Boehringer Mannheim reagent) was found satisfactory for two-point kinetic use on the Kone CD. The correlation of this method with the phosphotungstate reduction method used on the Technicon SMA 6/60 showed a large intercept (Boehringer Mannheim [$y$ axis], SMA [$x$ axis], $N=100$, $y=1{\cdot}042\ x-43{\cdot}1$). The day-to-day coefficients of variation for bovine control sera with low, medium, and high concentrations were 4·3%, 3·5%, and 2·5% respectively.

### Détermination cinétique à deux points par l'analyseur Kone CD d'acide urique dans un sérum avec uricase, catalase et aldéhyde dehydrogenase

G. A. Harff et F. Lamie

Deux méthodes usuelles pour la détermination enzymatique d'acide urique (d'après la nouvelle méthode ALDH de Häckel) sont examinées par rapport à leurs applications routinières avec l'analyseur Kone CD. Seul un réactif (celui de Boehringer, Mannheim) se présentait comme satisfaisant pour le cinétique à deux points avec le Kone CD. La corrélation de cette méthode avec la méthode par réduction avec acide phosphotungstique utilisé avec le Technicon SMA 6/60, monte une assez grande déviation du point d'intersection. (Boehringer Mannheim [axe $y$], SMA [axe $x$], $N=100$, $y=1{\cdot}042\ x-43{\cdot}1$). Le coefficient de variation des déterminations au jour le jour du sérum bovin de contrôle aux cencentrations petites, moyennes et grandes était de 4,3, 3,5% et 2,5%.

### Zwei-Punkt kinetische Bestimmung auf dem Kone CD Analysator von Harnsäure in Serum mit Urikase, Katalase und Aldahyd Dehydrogenase

G. A. Harff und F. Lamie

Zwei übliche Methoden für die enzymatische Bestimmung von Harnsäure nach Häckel's neuer ALDH-Methode werden im Bezug auf ihre Anwendbarkeit zu Routinezwecken auf dem Kone CD Analysator untersucht. Nur ein Reagens (das Boehringer Mannheim Reagens) stellte sich für die Zwei-Punkt kinetische Anwendung auf dem Kone CD als zufriedenstellend heraus. Die Korrelation dieser Methode mit der Phosphowolfram Reduktionsmethode, wie auf dem Technicon SMA 6/60 benützt, zeigte eine eher abweichende Schnittstelle (Boehringer Manneheim [$y$-Achse], SMA [$x$-Achse], $N=100$, $y=1{,}042\ x-43{,}1$). Die Variationskoeffizienten der Bestimmungen von Tag zu Tag für das Rind-Kontrollserum mit kleinen, mittleren und hohen Konzentrationen betrugen 4,3%, 3,5% und 2,5%.

### Forthcoming papers

Among the articles scheduled for the next issue of *Journal of Automatic Chemistry* are the following:

Assessment of the validity of error flags generated by the Technicon SMAC system and the Perkin-Elmer KA 150 enzyme analyser
*E. Arthur Robertson* et al.

A readily available source of serum for use as a precision quality-control material for ionized calcium measurement by ion-selective electrodes
*J. A. Fyffe*

A compact automated titration system, applied to a high-precision determination of calcium in sea-water
*L. Anderson and A. Granéli*

Automated determination of 5-hydroxyindolacetic acid in urine by high-performance liquid chromatography
*J. P. Garnier* et al.

